# Advances in the study of myeloid-derived suppressor cells in infectious lung diseases

**DOI:** 10.3389/fimmu.2023.1125737

**Published:** 2023-03-29

**Authors:** Meng-Nan Zhang, Yu-Lai Yuan, Su-Hua Ao

**Affiliations:** ^1^ College of Integrated Chinese and Western Medicine and the Affiliated Traditional Chinese Medicine Hospital, Southwest Medical University, Luzhou, Sichuan, China; ^2^ The Department of Respirology of the Affiliated Traditional Chinese Medicine Hospital, Southwest Medical University, Luzhou, Sichuan, China

**Keywords:** myeloid-derived suppressor cells, novel coronaviral pneumonia, tuberculosis, other infectious lung diseases, immunotherapy

## Abstract

Myeloid-derived suppressor cells (MDSCs) are a heterogeneous population of immature cells capable of inhibiting T-cell responses. MDSCs have a crucial role in the regulation of the immune response of the body to pathogens, especially in inflammatory response and pathogenesis during anti-infection. Pathogens such as bacteria and viruses use MDSCs as their infectious targets, and even some pathogens may exploit the inhibitory activity of MDSCs to enhance pathogen persistence and chronic infection of the host. Recent researches have revealed the pathogenic significance of MDSCs in pathogens such as bacteria and viruses, despite the fact that the majority of studies on MDSCs have focused on tumor immune evasion. With the increased prevalence of viral respiratory infections, the resurgence of classical tuberculosis, and the advent of medication resistance in common bacterial pneumonia, research on MDSCs in these illnesses is intensifying. The purpose of this work is to provide new avenues for treatment approaches to pulmonary infectious disorders by outlining the mechanism of action of MDSCs as a biomarker and therapeutic target in pulmonary infectious diseases.

## 1 Introduction

With the spread of novel coronavirus pneumonia (COVID-19), the resurgence of tuberculosis, and the emergence of antibiotic resistance in bacterial pneumonia, standard antiviral, anti-tuberculosis and antibiotic therapy against bacteria have reached a bottleneck. Immunotherapy for infectious illnesses has become one of the primary research foci as the hunt for therapies with fewer side effects and greater effectiveness has become imperative. In the inflammatory response to pathogens that assault the body and in the latter phases of chronic infection, the immune system is vital. Under normal circumstances, the innate immune system automatically recognizes and clears pathogens after they attack the body; however, when the innate immune system is compromised, pathogens are difficult to eliminate in a timely manner and are more likely to cause disease exacerbation or chronic infection. Myeloid-derived suppressor cells (MDSCs) are immunosuppressive cells, and the initial research on MDSCs focused mostly on malignancies, where MDSCs proliferate in the tumor microenvironment and may promote tumor proliferation and metastasis by mediating immune escape of tumor cells (1). Targeted immunotherapy against MDSCs promotes tumor regression by modulating the immune activity of T cells (2, 3). In addition, there is accumulating evidence that MDSCs also play a crucial role in regulating the immunological response of the body to infections. Numerous studies have also revealed that MDSCs may multiply and correlate with the severity of infectious lung disease, such as novel coronavirus pneumonia, tuberculosis, and bacterial pneumonia (4–10). Therefore, this research investigates the significance and mechanism of action of MDSCs in infectious lung illnesses, as well as MDSC immunotherapy.

## 2 Myeloid-derived suppressor cells

In the 1970s, a bone marrow-derived cell that suppressed T cells was identified in a mouse model of lung cancer and give the name “nature suppressor cells (NS)” due to its myeloid origin and immunosuppressive activity (11, 12). These cells were renamed “immature myeloid cells (IMCs)” or “myeloid suppressor cells (MSCs)” towards the beginning of the twenty-first century (13, 14). Due to the morphological, phenotypic, and functional heterogeneity of these cell populations, their nomenclature was controversial internationally until 2007, when they were unified as “myeloid-derived suppressor cells (MDSCs)” to describe heterogeneous cell populations of immature myeloid cells found in pathological settings (15). MDSCs are cells formed mostly from bone marrow hematopoietic precursor cells, which are progenitors of granulocytes, dendritic cells, or macrophages, and which are extensively dispersed in bone marrow, spleen, peripheral blood, cancers, and other tissues with significant heterogeneity and immunosuppressive activity (16, 17). Depending on their phenotypes, MDSCs may be divided into granulocytic/polymorphonuclear myeloid-derived suppressor cells (PMN-MDSCs or G-MDSCs) and monocytic myeloid-derived suppressor cells (M-MDSCs). MDSCs in mice are all expressed as CD11b and can be classified into PMN-MDSCs (CD11b^+^ Ly6C^low^ L6G^+^) and M-MDSCs (CD11b^+^ Ly6C^hi^ Ly6G^-^) according to the expression levels of Ly6G and Ly6C (5, 18). In mice chronically infected with Staphylococcus aureus, Eo-MDSCs (CD11b^+^ SyglecF^+^ CCR3^low^ IL-5Ra^low^ SSC-A^high^) with phenotypic characteristics of immature eosinophils were identified (16, 19). MDSC subtypes and phenotypic markers in human peripheral blood mononuclear cells (PBMC) include PMN-MDSCs (CD11b^+^ CD14^-^ CD33^+^ CD15^+^ HLA-DR^-/low^) and M-MDSCs (CD11b^+^ CD14^+^ CD33^+^ CD15^-^ HLA-DR^-/low^) (20, 21).

**Figure 1:**
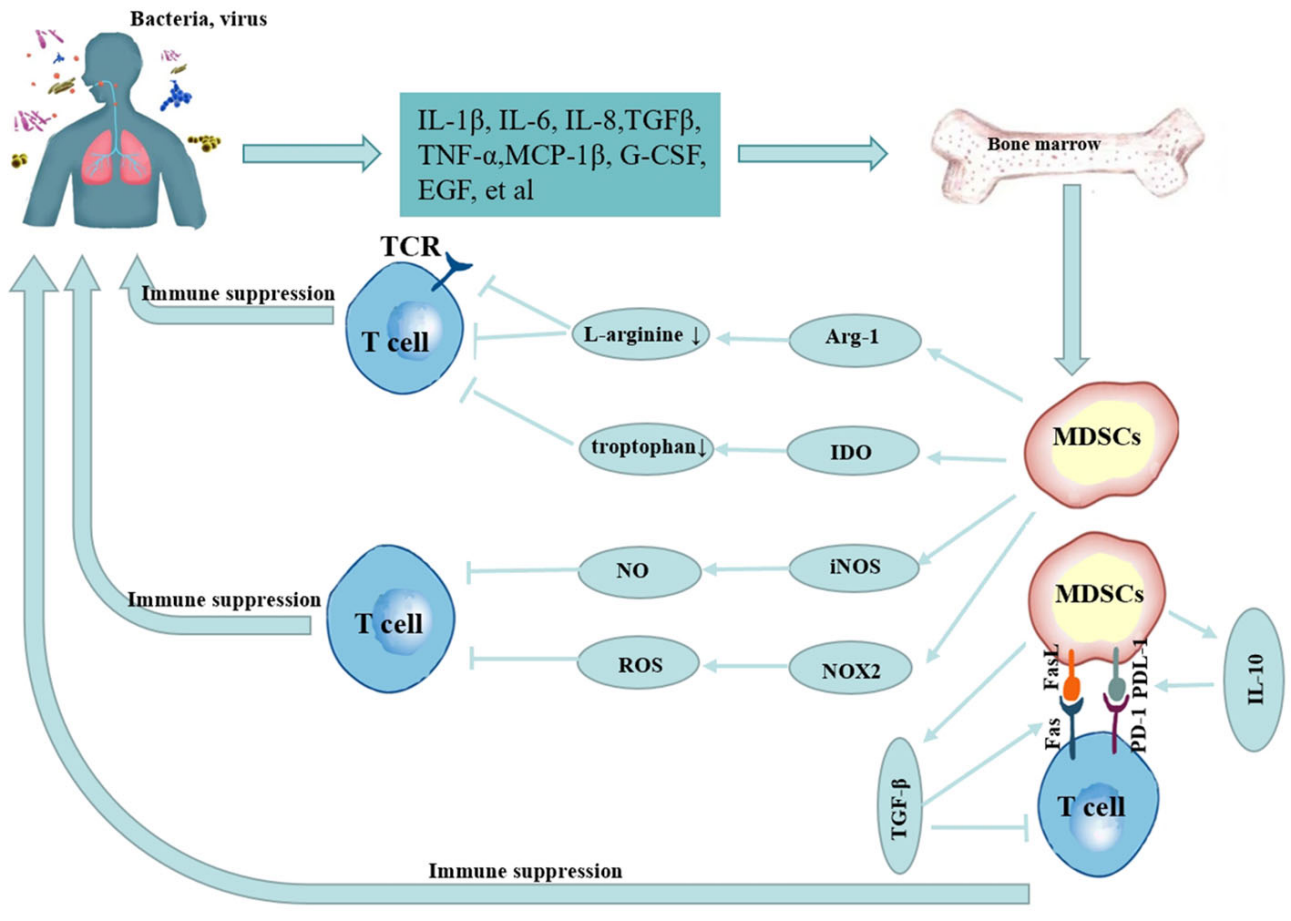
Mechanism of MDSCs in infectious lung diseases.

In a healthy state, immature myeloid cells may be produced in the bone marrow and develop into mature granulocytes, macrophages, or dendritic cells, which then penetrate the proper tissues and organs to execute typical immune tasks. However, their normal differentiation is hindered in pathological settings such as tumors, infectious diseases, and autoimmune diseases, and MDSCs can rapidly accumulate and be activated through injury-associated molecular patterns or pathogen-associated molecular patterns, etc., by promoting reactive oxygen species (ROS) release; expressing high levels of arginase-1 (Arg-1) (22) and inducible nitric oxide synthase (iNOS) (23), promoting the release of interleukin (IL)-10, IL-1β, IL-6, tumor necrosis factor (TNF)-α and other cytokine (10, 24–27), and the immunosuppressive activity of M-MDSCs was stronger than that of PMN-MDSCs (16, 28–30). The mechanism of MDSCs in infectious lung diseases is shown in **Figure 1**. Despite the fact that the majority of data on MDSCs are generated from malignancies, pulmonary infectious illnesses have commonalities with their activity in tumors and are linked to poor clinical outcomes (5, 31, 32). Nevertheless, in infectious lung disorders, the behavior of MDSCs in infections seems to be dependent on the kind of invading pathogen and the disease stage (30, 33).

## 3 MDSCs as a target for infectious lung diseases

### 3.1 Novel coronavirus pneumonia

Novel coronavirus pneumonia (COVID-19) remains a potentially life-threatening global pandemic acute infectious disease characterized by inflammatory storms, coagulation disorders, and organ damage (34). MDSCs with immunomodulatory activity were found to play an important role in mediating the excessive inflammation or inflammatory storm of COVID-19, and MDSCs can limit infection-induced excessive inflammation or inflammatory storm and protect host immunity (34); on the other hand, excessive inflammation or inflammatory storms also lead to accumulation of MDSCs in the peripheral blood of COVID-19 patients, participate in the pathological process of the disease, and correlate with the severity of the disease (8, 35–39). Significantly, a link between MDSCs and COVID-19 has been identified in a number of recent investigations (**Table 1**). Compared with healthy subjects or patients with mild COVID-19, the frequency of PMN-MDSCs in the peripheral blood of COVID-19 increases with disease exacerbation, especially in severe instances and deceased patients, and proliferating PMN-MDSCs will further suppress T cells, resulting in a reduction in lymphocytes and further compromising the host immune response, so establishing a vicious cycle (7, 20, 44, 45). Comparison with indicators reflecting disease severity (levels of C-reactive protein, ferritin, and lactate dehydrogenase) demonstrated that these indicators were elevated with PMN-MDSCs in severe cases, especially immature PMN-MDSCs, confirming a positive correlation between PMN-MDSCs and COVID-19 severity (41). A 1% increase in PMN-MDSC frequency was independently related with a 3% increase in the probability of fatal outcomes, as determined by an age- and gender-adjusted Cox regression model (20). In contrast to these results, Japanese researchers found that the frequency of PMN-MDSCs (but not other MDSC subgroups) may be transiently elevated in patients surviving severe COVID-19 compared to patients dying from severe COVID-19, and the investigators suggest that PMN-MDSCs may reduce detrimental immune responses and be associated with genetic factors (36). In severely sick individuals, low levels of PMN-MDSCs may aid in survival (45). However, the majority of investigations have revealed that the frequency of M-MDSCs in peripheral blood following COVID-19 therapy is substantially linked with disease severity (8, 37). Analysis of SARS-CoV-2 viral RNA burden revealed a connection between M-MDSCs and viral load, indicating that SARS-CoV-2 infection may inhibit host immunological responses by encouraging the proliferation of M-MDSCs (43). M-MDSCs inhabit CD4+ and CD8+ T cell proliferation and IFN-γ production through an Arg-1-dependent mechanism, with downregulation of CD3ζ chain expression (8). Despite the fact that MDSCs and cytokine levels (such as IL-6, TNF, IL-1, etc.) remained persistently elevated during the recovery phase of COVID-19, this also suggests that MDSCs exerted an inhibitory T-cell recall response, that the suppressive activity of T cells persisted after recovery from infection (39, 42). It can be seen that M-MDSCs not only suppress the immune activity of T cells during the acute episode after SARS-CoV-2 infection, but also have a recall response to suppress T cells during the recovery period. In conclusion, the results of different studies may vary, but MDSCs exhibit different phenotypic characteristics and functional status with various stages of COVID-19, and MDSCs, as one of the key pathogenic factors of COVID-19 inflammation and immunosuppression, may be a major target for treatment (30, 46, 47).

**Table 1:** Summary current studies on MDSCs in COVID-19 patients.

Samples (COVID-19 patients and healthy donors (HD))	Source of MDSCs	Subtypes and phenotypic markers of MDSCs	Frequency of MDSCs	Cytokines	Reference
N=68(COVID-19:48; HD:20)	PBMC	CD11b,CD14,HL (39)A-DR,CD33,CD88,CD56,CD19,CD3,CD15,CD45,DRAQ7	MDSCs↑ (as compared to HD)	IL-1β, IL-6, IL-8 and TNF-α↑	(40)
N=68(COVID-19:15 severe, Nosevere:26; HD:26)	PBMC	Total MDSCs: HLA-DR^-^CD11b^+^; Mature PMN-MDSCs: HLA-DR^-^CD11b^+^CD15^+^CD66b^+^CD14^-^CD16^+^; Immature PMN- MDSCs: HLA-DR^-^CD11b^+^CD15^+^CD66b^+^CD14^-^CD16^-^; M-MDSCs: HLA-DR^-^CD11b^+^CD15^-^ CD66b^-^CD14^+^	Total MDSCs, mature PMN-MDSCs, immature PMN- MDSCs and M-MDSCs↑ (as compared to HD); mature PMN-MDSCs and immature PMN- MDSCs severe patients (as compared to nosevere)	None	(41)
N=22(CoV2-=9, CoV2+=13) SARS Coronavirus 2	PBMC	M-MDSCs;CD11b^+^CD33^+^CD14^+^HLA- DR^-/lo^	M-MDSCs↑	IL-6↑	(39)
N=66(COVID-19 = 56, moderate: 45, severe: 11; HD=10); after 3 months (n=21)	EDTA-anticoagulated blood	PMN-MDSCs: CD11b^+^CD14^-^CD15^+^CD16^+^CD33^-^HLA-DR^-^; M-MDSCs: CD11b^+^CD14^+^CD15^-/low^CD16^-^CD33^+^HLA-DR^-/low^	PMN-MDSCs: severe: moderate=10:1; M-MDSCs: severe: moderate=4:1	IL-1β, IL-6, IL-7, EGF, HCF, PDGF-BB, et al. ↑	(42)
N=158(COVID-19 = 96(ICU:32, non-ICU:96); HD:30); 59 patients recovered; 19 died	PBMC	PMN-MDSCs: CD11b^+^CD14^-^CD33^+^CD15^+^ HLADR^-/low^; M-MDSC: CD11b^+^CD14^+^CD33^+^HLA-DR^-/low^	PMN-MDSCs↑,especially in patients who required intensive care treatments (as compared to HDs)	IL-1β, IL-6, IL-8, and TNF-α↑	(20)
N=40(COVID-19 = 27(ICU:8, non-ICU:19; HD:13)	EDTA-anticoagulated peripheral blood	PMN-MDSCs: CD11b^+^CD33^+^CD15^+^HLA-DR^+^; M-MDSCs: CD11b^+^CD33^+^CD14^+^HLA-DR^lo/neg^ or CD14^+^HLA-DR^lo/neg^; the new subset of MDSCs: CD14^+^HLA-DR^lo/neg^	CD14^+^HLA-DR^lo/neg^ MDSCs↑, especially in patients who required intensive care treatments (as compared to HD)	None	(43)
N=71(COVID-19 = 62(ICU:31, non-ICU:31; HD:9))	PBMC	PMN-MDSCs: HLA-DR^low/-^ CD11b^+^CD14^-^CD33^+^CD15^+^; M-MDSCs: HLA-DR^low/-^CD11b^+^ CD14^+^CD33^+^	PMN-MDSCs↑, especially in patients who required intensive care treatments (as compared to HD)	None	(44)
N=26(COVID-19 = 18(mild:9; severe:9); HD=8)	PBMC	PMN-MDSCs: HLA-DR^-^Lin^-^CD33^+^CD11b^+^CD15^+^	PMN-MDSCs↑, especially in severe (as compared to HD)	IL-6, IL-8, IL-1β, TNF-α↑; TGF-β↑ (in plasma from patients with severe disease, which decreased over time.)	(7)
N=224 (COVID-19 = 147; influenza A virus infected patients=44; HD=33)	PBMC and nasopharyngeal Aspirates (NPA)	M-MDSCs : CD14^+^ Lin^-^(CD3^-^CD56^-^CD19^-^CD20^-^CD66^-^) HLA-DR^-^; PMN-MDSCs: CD56^-^CD14^-^CD3^-^CD19^-^HLA-DR^-^CD66abce LOX-1	M-MDSCs↑ (as compared to influenza A virus infected patients and HDs)	IL-6, IL-10↑	(8)
N=80 (COVID-19 with ICU treatment)	Whole blood samples	M-MDSCs: CD45^+^CD11b^+^CD33^+^HLA-DR^low/−^ CD14^+^CD15^-^, G-MDSCs: CD45^+^CD11b^+^CD33^+^HLA-DR^low/-^ CD14^-^CD15^+^	PMN-MDSCs ↓ (as compared to the ICU deceased)	None	(45)
N=47(COVID-19 = 40(mild:12; moderate I:7; moderate II:8; severe:13); HD=7)	PBMC	e-MDSCs : CD3^-^CD19^+^CD56^-^HLA-DR^-^CD11b^+^CD33^+^CD14^-^CD15^-^; M-MDSCs: D3^-^CD19^-^CD56^-^HLA-DR^-^CD11b^+^CD33^+^CD14^+^CD15^-^; PMN-MDSCs: CD3^-^CD19^-^CD56^-^HLA-DR^-^CD11b^+^CD33^+^CD14^-^CD15^+^	PMN-MDSCs ↑in survivors of severe COVID-19 (as compared to mild, moderate, deceased and HDs)	IL-8↑	(36)
N=32(excluded n=3; COVID-19 n=29(no secondary infection=17; secondary infection:12))	EDTA anticoagulated blood	M-MDSCs: CD11b^+^HLA-DR^-^	M-MDSCs↑	None	(31)
N=57(COVID-19 = 41(mild:21; severe:20); HD=16)	PBMC	PMN-MDSCs: Lin^-^HLA-DR^low^CD11b^+^SSC^low^CD15^+^CD66b^+^; M-MDSCs: Lin^-^HLA-DR^low^CD11b^+^SSC^low^CD14^+^; e-MDSCs: Lin^-^HLA-DR^low^CD11b^+^SSC^low^CD15^-^CD66b^-^/CD14^-^	PMN-MDSCs and M-MDSCs↑ (as compared to HDs)	IL-6,IL-8,MCP-1,IL-18, TGF-β, IL-10↑	(38)
N=26(ARDS COVID: 13; Moderate COVID:13)	PBMC	M-MDSCs: CD14^+^HLA-DR^-^; PMN-MDSCs: CD45^+^Lin^-^HLA-DR^-^CD15^+^CD11b^+^; e-MDSCs : CD45^+^CD3^-^Lin^-^HLA-DR^-^CD15^+^CD33^+^	M-MDSCs and PMN-MDSCs↑	IL-6, IL-10, MCP-1, CXCL9, CXCL10, ↑(as compared to HDs),and G-CSF↑ in ARDS	(37)

↑ represents increased, ↓ represents decreased, PBMC, peripheral blood mononuclear cell; IL-1β, interleukin-1β; IL-6, interleukin-6; IL-8, interleukin-8; IL-10, interleukin-10;IL-18, interleukin-18;TNF-α, tumor necrosis factor-α; TGF-β, transforming growth factor-β; EGF, Epidermal Growth Factor; PDGF-BB, Platelet derived growth factor-BB; MCP-1, monocyte chemotactic protein-1; G-CSF, granulocyte-colony stimulating factor; CXCL9, chemokine (C-X-C motif) ligand 9; CXCL10, chemokine (C-X-C motif) ligand 10.

Inflammatory cytokines are one of the primary mechanisms that induce expansion of MDSCs and are the focus of research on COVID-19-targeted therapy (40, 48). The pro-inflammatory cytokine IL-6 phosphorylates STAT3 *via* the gp130/JAK/STAT pathway, hence regulating M-MDSC differentiation, proliferation and survival in human and animal disease conditions including COVID-19 (49–51). In addition, studies have shown that IL-6 levels rise with the degree of illness in individuals with severe disease (7, 8, 52). Therapeutic modulation of IL-6 levels by anti-IL-6 receptor antagonists (tocilizumab, sarilumab) reduces the duration of COVID-19 and/or reduces the severity of the disease (53–55). *In vitro* culture of PBMC isolated from peripheral blood of COVID-19 patients revealed that 5-fluorouracil (5-FU) restored lymphocyte proliferation and propagated Th1-mediated immune response by decreasing levels of MDSCs and decreasing production of IL-10, IL-8, IL-17, and Th2 cytokines, while boosting production of IFN-γ and IL2 (38). In COVID-19 therapy, it has been proposed that 5-FU in conjunction with deoxyribonucleosides and deoxyribose may have antiviral effects (56). In addition, it has also been hypothesized that vitamin D deficiency increases the risk of developing ARDS in COVID-19 patients and that vitamin D supplementation may attenuate the inflammatory response caused by pulmonary macrophages and MDSCs in COVID-19 patients and reduce acute respiratory distress syndrome in COVID-19 patients (57). Despite the fact that the aforementioned studies demonstrated that targeting MDSCs for the treatment of COVID-19 may be more effective, these studies are still restricted to *in vitro* cell culture and clinical trials, and the particular therapeutic processes need more research.

### 3.2 Tuberculosis

Tuberculosis is a devastating infectious disease caused by Mycobacterium tuberculosis, as of the year 2020, it has superseded SARS-CoV-2 as the second most infectious disease killer, with roughly 1.3 million fatalities every year (58). Although BCG vaccination and antituberculosis chemotherapy have been extensively utilized for TB prevention and treatment, the consequences have been unsatisfactory (6, 59), thus, it has become necessary to investigate alternative antituberculosis treatment strategies. MDSCs have been found to make a significant contribution in the pathology of TB, and the majority of studies indicate that MDSCs provide ecological niches for the survival of Mycobacterium avium in the lungs of infected hosts and promote replication of Mycobacterium tuberculosis at the site of pulmonary infection (5, 6, 60, 61). Recent investigation has shown that Mycobacterium tuberculosis may employ the MPT64 protein to stimulate the creation of MDSCs, hence facilitating its survival and evasion of host immunological defenses (62). MDSCs not only accumulate in the peripheral blood of M. tuberculosis model mice (63), but also in the spleen of M. tuberculosis-infected mice (64). In addition, an increased frequency of MDSCs was observed in peripheral blood, bronchoalveolar lavage fluid, and pleural fluid specimens from patients with pulmonary or extrapulmonary tuberculosis, and the frequency of circulating MDSCs also decreased significantly at the end of antituberculosis treatment, indicating that MDSCs play an important role in the pathogenesis of tuberculosis (6, 65–69). *In vitro* granuloma model tests have shown that human MDSCs activate MAPK channels, hence boosting IL-1O production and Mycobacterium tuberculosis replication (60). The results of studies on TB patients also confirm the correlation between MDSCs and disease (**Table 2**). The frequency of both subpopulations of MDSCs was elevated in PBMC of patients with active TB, was dominated by M-MDSCs, reduced the immunological function of lymphocytes in TB patients, and was proportional to the severity of the disease (67–70). Other investigations have shown that the levels of PMN-MDSCs are elevated in the peripheral blood and bronchoalveolar lavage fluid of patients with active TB, and that these levels correlate with plasma nitric oxide levels (6, 72). Grassi et al. established the link between PMN-MDSCs and TB severity by confirming by chest X-ray and experiment that PMN-MDSCs levels were higher in patients with milder disease severity than in those with more severe disease severity (71). Bindu et al. further found through studies on non-human primate TB granulomas that PMN-MDSCs levels were elevated in animal models of active TB (ATB) compared to latent TB-infected animals and were located in the lymphocyte cuffs surrounding the granuloma, thereby inhibiting T-cell entry into granuloma’s core (73). These results indicate that MDSCs may represent a novel target for TB host-directed treatment and a possible signal for detecting success.

**Table 2:** Summary current studies on MDSCs in tuberculosis patients.

Samples	Source of MDSCs	Subtypes and phenotypic markers of MDSCs	Frequency of MDSCs	Cytokines	Reference
N=62(TB=43(low responders (LR-TB):23, high responders (HR-TB):20); HD=19)	PBMC	M-MDSCs: CD14^+^CD33^+^CD11b^+^CD15^-^HLA-DR^-/low^; PMN-MDSCs: CD15^+^CD33^+^CD11b^+^CD14^-^HLA-DR^-/low^	M-MDSCs and PMN-MDSCs ↑ (M-MDSCs ↑ in LR-TB; PMN-MDSCs ↑ in HR-TB)	IFN-γ↓ (in LR-TB)	(70)
N=48 (active TB=38; HD=10)	PBMC	M-MDSCs: Lin^-^HLA-DR^-/low^CD33^+^CD11b^+^CD14^+^CD15^-^; PMN-MDSCs: Lin^-^HLA-DR^-/low^CD33^+^CD11b^+^CD14^-^CD15^+^; e-MDSCs: Lin^-^ (CD3/CD14/CD15/CD19/56)HLA-DR^-^CD33^+^	M-MDSCs↑ (as compared to recovered and HDs)	IL-6 ↑	(67)
N=45 (active TB=35; HD=10)	PBMC and bronchoalveolar cells (BALc)	PMN-MDSCs: CD11b^+^CD14^-^CD33^+^CD15^+^HLA-DR^low^; M-MDSCs : CD11b^+^CD14^+^CD33^+^ HLA-DR^low^; MDSCs: HLA-DR^-/low^CD11b^+^CD33^+^	MDSCs ↑ (in PBMCs and BALc); PMN-MDSCs ↑ (as compared to HDs)	None	(6)
N=230 (active TB=110; latent TB infection (LTBI)=80; HD=40)	PBMC	PMN-MDSCs: CD14^-^CD15^+^CD11b^+^CD33^+^HLA-DR^low/−^; M-MDSCs: CD14^+^CD15^-^CD11b^+^CD33^+^HLA-DR^low/-^; MDSCs: CD33^+^ HLA-DR^-/LOW^	MDSCs↑ (as compared to HDs)	IFN-γ↓	(68)
active TB and household contacts (HHC)	PBMC and/or pleural fluid	MDSCs: LIN^-/lo^ HLA-DR^+^CD33^+^CD11b^+^; M-MDSCs: HLA-DR^-/lo^CD11b^+^CD14^+^ or S100A9^+^; PMN-MDSCs: HLA-DR^-/lo^CD11b^+^CD15^+^	MDSCs↑	IL-1β, IL-6, IL-8, G-CSF,MCP-1↑; GM-CSF and MIP-1β↓	(65)
N=16 (standard TB treatment=8; standard TB treatment+ COX-2i=8);	PBMC	NDSCs: HLA-DR^neg/low^CD14^+^CD33^+^CD11b^+^	M-MDSCs↑	None	(69)
N=49 (active TB=19; latent TB infection (LTBI)=18; HD=12)	PBMC	PMN-MDSCs: HLA-DR^-/low^CD11b^+^CD14^-^CD15^+^/CD66b^+^; M-MDSCs: HLA-DR^-/low^CD11b^+^CD33^+^ CD14^+^CD15^-^; e-MDSCs: HLA-DR^-^CD33^+^CD15^-^Lin(CD3^-^CD56^-^CD19^-^CD14^-^)	PMN-MDSCs ↑ (as compared to LTBI and HDs)	None	(71)
N=33 (active TB=23; latent TB infection (LTBI)=10)	PBMC	e-MDSCs: LIN1^-^HLA-DR^-/low^CD11b^+^CD33^+^; PMN-MDSCs: HLA-DR^-/low^CD14^-^CD15^+^CD33^+/dim^; M-MDSCs: HLA-DR^-/low^CD14^+^CD15^-^CD33^+^	PMN-MDSCs↑ (as compared to LTBI)	None	(72)

IFN-γ, interferon-γ; IL-1β, interleukin-1β; IL-6, interleukin-6; IL-8, interleukin-8; G-CSF, granulocyte-colony stimulating facto; GM-CSF, granulocyte-macrophage colony stimulating factor; MCP-1, monocyte chemotactic protein-1; MIP-1β, macrophage inflammatory protein 1β.

↑ represents increased and ↓ represents decreased.

Host-directed therapy (HDT) is a novel approach to innovative host-specific therapies designed to reduce excessive inflammation or enhancing the host’s immune defense against pathogens, with the goal of shortening treatment regimens without inducing drug resistance (74). Several FDA-approved medicines, including all-trans retinoic acid (ATRA), cyclooxygenase-2 inhibitor (COX-2i), phosphodiesterase-5 inhibitor (PDE-5i), and sildenafil, have been validated in the treatment of tuberculosis (TB) (61, 69, 74–76). It was found that MDSCs levels were excessively elevated in the lungs of a mouse model of tuberculosis, which was related with increased mortality, and the frequency of MDSCs decreased while the number of T cells rose after host-directed therapy with all-formic retinoic acid (ARTA) (61). COX-2i has been demonstrated to reduce pathological lung damage caused by the host immunological response in tuberculosis patients (76). Combining COX-2i with anti-inflammatory effects with anti-tuberculosis basal treatment reduced cytokines that induce high levels of M-MDSCs, including IL-1, IL-10, IL-6, TNF, and S100A9 (69). Combining PDE-5i sildenafil with antituberculosis basal therapy improved treatment efficacy because PDE-5i sildenafil increased cyclic adenosine monophosphate (cGMP) in MDSCs, leading to a decrease in Arg-1 and nitric oxide synthase 2 (NOS2), thereby decreasing the mechanism of MDSCs-induced T-cell suppression (74). However, Vinzeigh N et al. demonstrated that sildenafil was incapable of reversing MDSCs-mediated T-cell suppression and had little effect on enhancing host immunity (77). The above findings for MDSCs-targeted therapy suggest that MDSCs may be a new target for anti-tuberculosis host-directed therapy, but the results are contradictory and additional investigation is required.

### 3.3 Other infectious lung diseases

Studies on the correlation between MDSCs and infectious lung diseases have included lung injury caused by pathogens such as Streptococcus pneumoniae, Staphylococcus aureus, Klebsiella pneumoniae, Pseudomonas aeruginosa, and Pneumocystis carinii, in addition to the two specific pathogens mentioned above. In a mouse model of Streptococcus pneumoniae pneumonia, MDSCs levels are elevated in model mice’s circulation and are associated with a choline-binding protein, although the specific mechanism remains unclear (78). However, the results of an animal experiment combined with clinical trials suggest that the macrolide antibiotic clarithromycin promotes elevated levels of MDSCs in the circulation of animals and humans through a particular mechanism that promotes the expansion of MDSCs (CD11b+Gr-1+) through the STAT3/Bv8 axis, decreases INF-γ, boosts IL-10 levels, and protects the organism from post-influenza Streptococcus pneumoniae infection (79). Staphylococcal enterotoxin B secreted by Staphylococcus aureus induces an increase in circulating levels of MDSCs in Staphylococcus aureus-infected mice, and treatment with resveratrol increases the proportion of circulating MDSCs because MDSCs can downregulate the body’s immune response to prevent tissue damage at the site of inflammation (80). MDSCs play a pivotal part in the efferocytosis of neutrophils following infection with Klebsiella pneumoniae, and elevated levels of MDSCs in animal models of Klebsiella pneumoniae pneumonia promote IL-10 production in the late stages of infection to facilitate the efferocytosis of apoptotic neutrophils and reduce lung injury (10, 81). It has also been hypothesized that the early expansion of M-MDSCs during an infection terminates the proinflammatory signaling essential for the clearance of Klebsiella pneumoniae, hence causing a chronic infection (82). While the particular mechanism of MDSCs in Klebsiella pneumoniae pneumonia remains a complicated process, it is known that MDSCs are involved (83). Both in clinical trials and in animal studies, P. aeruginosa infection leads to increased levels of circulating PMN-MDSCs in patients with chronic inflammatory diseases of the lung, including pulmonary cystic fibrosis, disrupting the host immune response (84). Levels of MDSCs in alveolar lavage fluid in animal models of Pneumocystis pneumonia (PcP) increase with increasing numbers of Pneumocystis carinii in the organism and with increasing lung inflammation; moreover, secondary transfer of MDSCs may directly cause lung damage in normal mice (85). Treatment with immunosuppressive drugs and antibiotics (all-trans retinoic acid combined with Primaquine) transforms MDSCs in the lung into alveolar macrophages capable of clearing Pneumocystis infection, enabling the host to successfully fight against infection (86). Further studies revealed that MDSCs are depleted of alveolar macrophage phagocytic activity during PcP *via* the PD-1/PD-L1 pathway (87). Despite the fact that the majority of the aforementioned studies on other pulmonary infectious diseases are limited to animal experiments, the fact that MDSCs are associated with the disease, for better or for worse, regardless of the type of pulmonary infectious disease may indicate that MDSCs are poised to become another therapeutic target for these diseases.

## 4 The role of traditional Chinese medicine in infectious lung diseases

Traditional Chinese medicine (TCM) refers to traditional medicine that studies the relationship between human physiology and pathology and the natural environment from a dynamic and holistic perspective under the guidance of the theory of yin-yang and the five elements, and explores effective methods to prevent and treat diseases, with a holistic view and discriminatory treatment as its main ideas. As traditional medicine, TCM is an important part of the medical field with a long history and rich experience in preventing and treating infectious diseases. Modern research results have also demonstrated the advantages of TCM in improving clinical symptoms, suppressing pathogens, promoting host immunity, and reducing side effects (88). Particularly, during COVID-19 pandemic, the vast majority of novel coronavirus pneumonia patients in China received TCM treatment, showing that TCM can significantly alleviate symptoms, reduce the inflammatory response, and promote recovery in patients with novel coronavirus pneumonia (89, 90). Several findings involving methods such as network pharmacology and molecular docking techniques have also pointed out that the active ingredients of single herbal medicines such as glycyrrhiza, scutellaria baicalensis, Coptis chinensis and lonicera japonica, and compound herbal medicines such as Yinqiaosan and LianhuaQingwen capsule can act on different targets and pathways of COVID-19, such as angiotensin-converting enzyme 2 (ACE2), TNF signaling pathway, T-cell receptor signaling pathway, Toll-like receptor signaling and MAPK signaling pathway (91–95). *In vitro* experiments have also demonstrated that LianhuaQingwen capsule inhibits the replication of SARS-CoV-2 and significantly reduces the production of pro-inflammatory cytokines (TNF-α, IL-6, CCL-2/MCP-1 and CXCL-10/IP-10) (96). In tuberculosis, the active ingredients of TCM not only modulate the cellular immune function of the body and promote the clearance of Mycobacterium tuberculosis, but also play a role in suppressing the inflammation of the body and inhibiting the development of drug resistance in Mycobacterium tuberculosis, such as gynostemma pentaphylla, luteolin and isoliquiritigenin (97–101). In addition, in pneumonia infected with P. aeruginosa, the active ingredients of TCM active ingredients not only inhibit the release of cytokines and chemokines in the organism, such as TNF-α, IL-6, IL-4, IL-8, and RANTES, to improve the lung infection, but also may inhibit the proliferation of P. aeruginosa through PI3K/AKT and Ras/MAPK pathways, selectively act on the QS (quorum sensing) of P. aeruginosa system to reduce bacterial virulence, and inhibition of P. aeruginosa biofilm formation (102–104).

## 5 Conclusions

MDSCs were discovered for the first time in oncological disorders, where they play unique immunomodulatory functions under pathological settings. There are increasing indications that MDSCs play a crucial role in regulating the immunological response of the organism, particularly in lung infectious illnesses. Depending on the disease state and research methodology, the percentage of MDSC subtypes might vary. In spite of the fact that the subtypes and levels of MDSCs in the host correlate with the severity of the disease, the exact mechanism of action of MDSCs in various diseases is still a matter of debate. MDSCs function as an immunosuppressive cell that inhibits the acute inflammatory response, promotes inflammation subsidence, and initiates the repair process of the organism, thereby ameliorating clinical symptoms, such as those caused by Streptococcus pneumoniae pneumonia and staphylococcal enterotoxin infection pneumonia. In COVID-19, tuberculosis, Pseudomonas aeruginosa infection, and Pneumocystis pneumonia, however, the levels of MDSCs in the circulation are positively correlated with the degree of inflammation of the disease, as MDSCs further impair the host’s immune response, resulting in persistent and recurrent bacterial or viral infections. In conclusion, there is a correlation between MDSCs and a variety of pulmonary infectious diseases, and the findings suggest that targeting MDSCs may reduce adverse drug reactions and resistance, and that MDSCs would be one of the important targets in the treatment of these pulmonary infectious diseases, with immune-targeted therapy against MDSCs being clearly proposed in the treatment of tuberculosis. These investigations imply that MDSCs create a pivotal regulatory function in lung infectious illnesses; nevertheless, due to the complexity of the disease, focused treatment in contemporary medicine has not yet been able to modify the disease’s overall development environment.

TMC, a traditional medicine with a holistic view and evidence-based treatment as the main ideas, is an important part of the medical field with a long history and rich experience in the prevention and treatment of infectious diseases. TMC plays a role in COVID-19, tuberculosis and other pulmonary infectious diseases by improving clinical symptoms, inhibiting pathogen proliferation, promoting pathogen clearance, regulating host immunity, reducing adverse effects and inhibiting pathogen resistance, highlighting the multi-target advantages of Chinese medicine in pulmonary infectious diseases. It can be seen that TCM may be an important available resource to target MDSCs for the treatment of pulmonary infectious diseases. There are no studies that have employed TCM to modify MDSCs and thereby affect lung infectious illnesses, according to a review of a broad body of research. Consequently, our future research will focus on advancing TCM research into the investigation of MDSCs in lung infectious illnesses. In addition, it is essential to integrate TCM with contemporary medicine in order to maximize the benefits of TCM in increasing and lowering toxicity, as well as in treating both symptoms and underlying causes in order to enhance patients’ quality of life.

## Author contributions

M-NZ contributed to manuscript research and writing. Y-LY contributed to manuscript writing and review. S-HA contribute to manuscript supervision, writing, and review. All authors contributed to the article and approved the submitted version.
